# Empirical Formulas for Estimating Backscattering and Absorption Coefficients in Complex Waters from Remote-Sensing Reflectance Spectra and Examples of Their Application

**DOI:** 10.3390/s19184043

**Published:** 2019-09-19

**Authors:** Sławomir B. Woźniak, Mirosław Darecki, Sławomir Sagan

**Affiliations:** Institute of Oceanology, Polish Academy of Sciences, ul. Powstańców Warszawy 55, 81-712 Sopot, Poland

**Keywords:** empirical formulas, seawater inherent optical properties, backscattering and absorption coefficients, remote-sensing reflectance, hue angle, trichromatic colour vision, semi-empirical algorithms

## Abstract

Many standard methods used for the remote sensing of ocean colour have been developed, though mainly for clean, open ocean waters. This means that they may not always be effective in complex waters potentially containing high concentrations of optically significant constituents. This paper presents new empirical formulas for estimating selected inherent optical properties of water from remote-sensing reflectance spectra *R_rs_*(*λ*), derived, among other things, for waters with high concentrations of dissolved and suspended substances. These formulas include one for estimating the backscattering coefficient *b_b_*(620) directly from the magnitude of *R_rs_* in the red part of the spectrum, and another for estimating the absorption coefficient *a*(440) from the hue angle *α*. The latter quantity represents the water’s colour as it might be perceived by the human eye (trichromatic colour vision); it is easily calculated from the shape of the *R_rs_* spectrum. These new formulas are based on a combined dataset. Most of the data were obtained in the specific, optically complex environment of the Baltic Sea. Additional data, taken from the NASA bio-Optical Marine Algorithm Dataset (NOMAD) and representing various regions of the global oceans, were used to widen the potential applicability of the new formulas. We indicate the reasons why these simple empirical relationships can be derived and compare them with the results of straightforward modelling; possible applications are also described. We present, among other things, an example of a simple semi-analytical algorithm using both new empirical formulas. This algorithm is a modified version of the well-known quasi-analytical algorithm (QAA), and it can improve the results obtained in optically complex waters. This algorithm allows one to estimate the full spectra of the backscattering and absorption coefficients, without the need for any additional a priori assumptions regarding the spectral shape of absorption by dissolved and suspended seawater constituents.

## 1. Introduction

As part of a discipline colloquially referred to as ocean colour remote sensing, different algorithms have been developed that permit the retrieval of a variety of information about the aquatic environment, based on satellite observations of light emerging from the water surface. The progress that has taken place in this field of science in the last few decades is documented, among others, in the reports issued by the International Ocean Color Coordinating Group (see [1] and earlier reports). One of the basic quantities spectrally describing the light emerging from water is the remote sensing reflectance *R_rs_*(*λ*), defined as the ratio of the water-leaving radiance to the downward irradiance (for precise definitions of the optical quantities, see e.g., the monograph by Mobley [2]). Many algorithms have been developed with which various biogeochemical properties of the surface water layer can be estimated directly from *R_rs_* spectra, e.g., the concentration of the main phytoplankton pigment chlorophyll *a*, or the concentration of particulate organic carbon, to name but a few. There is also another group of algorithms with which the so-called inherent optical properties (IOPs) of water can be estimated (see e.g., [3]). IOPs, by definition, are optical properties that do not depend on changes in the light fields in the atmosphere and within the water, and they generally describe how light can be absorbed and scattered by different constituents of the complex medium that is seawater. Importantly, IOPs can form a physically justified “link” between the remotely observed reflectance of the sea and different biogeochemical characteristics of individual seawater components.

An overview of the various possible approaches for retrieving marine IOPs from remote sensing can be found, for example, in the recent work by Werdell et al. [4]. It is generally known that, owing to the complexity of the formation of the upwelling light field in water, the algorithms for retrieving IOPs cannot be reduced to purely analytical solutions: IOP algorithms always have to contain a certain dose of empiricism. Purely empirical solutions are possible, as are look-up table approaches using results of forward models, and solutions jointly referred to as semi-analytical inversions. Among the latter group, there are a number of distinct classes, one of which is the “spectral deconvolution” class (for details, see Werdell et al. [4]). This class is of special interest to the authors of this work. As opposed to other semi-analytical approaches, spectral deconvolution methods allow one to separate the process of estimating total seawater IOPs from the process of decomposing them into component spectra. A widely known example of a spectral deconvolution algorithm, is the quasi-analytical algorithm (QAA) developed by Lee et al. [5]. Although the original version of this algorithm was developed almost two decades ago, its updated versions are still frequently used by the ocean colour science community (e.g., by NASA’s Ocean Biology Processing Group [6], or by ESA’s Ocean Colour Climate Change Initiative project [7]).

Typical conditions in the waters of the Baltic Sea differ significantly from those in open oceanic regions. The Baltic Sea is an example of waters belonging to the broad category referred to as Case 2 according to the classification introduced by Morel and Prieur [8]. In this sea, there are usually very high concentrations of chromophoric dissolved organic matter (CDOM) [9], not correlated with the content of autogenic chlorophyll *a*, and variable concentrations of suspended substances [10], often much higher than in oceanic waters. Also, significant seasonal changes in phytoplankton absorption properties have been documented in this sea [11]. As a result of the composition of this seawater, *R_rs_* spectra recorded in the Baltic Sea have maxima clearly shifted towards red wavelengths compared to typical oceanic spectra [12,13]. Optical and bio-optical relationships developed mainly on the basis of data from open oceanic regions are often inapplicable to Baltic waters. This is the case, for example, with standard algorithms estimating chlorophyll *a* concentration [14]. As we will show in the example analysed at the end of this work, this also happens when one attempts to estimate selected IOPs.

In addition to the quantities commonly used in ocean colour remote sensing, in this work we will also refer to the quantity known as the hue angle. It is a single parameter that can mathematically represent the colour of water as perceived by the human eye, which, as we know, uses the so-called trichromatic colour vision mechanism. The hue angle may also be associated with the classic colour scale of sea water, the so-called Forel-Ule scale, commonly used by oceanographers long before the era of precise spectral radiometers, including satellite radiometers. In recent years, there has been an evident resurgence of interest in the re-use of this scale and the historical data related to it [15,16,17,18,19,20,21,22,23,24].

The main aim of the current work was formulated as follows: to employ data collected in the specific conditions of the Baltic Sea as a basis for finding new forms of simple empirical formulas. These formulas should enable selected water IOPs to be estimated directly on the basis of remote-sensing reflectance spectra, especially in waters with high concentrations of dissolved and suspended substances. There was also an additional goal: to give examples of the possible use of the new formulas as calculation steps in new variants of semi-analytical algorithms for the retrieval of full spectra of seawater IOPs in the visible spectral range. These examples are intended to be an alternative to other known standard algorithms belonging to the “spectral deconvolution” class (like the QAA), which permit water IOPs to be retrieved without the need to adopt additional a priori assumptions regarding the spectral shapes of absorption coefficients by various seawater components.

## 2. Materials and Methods

**Figure 1:**
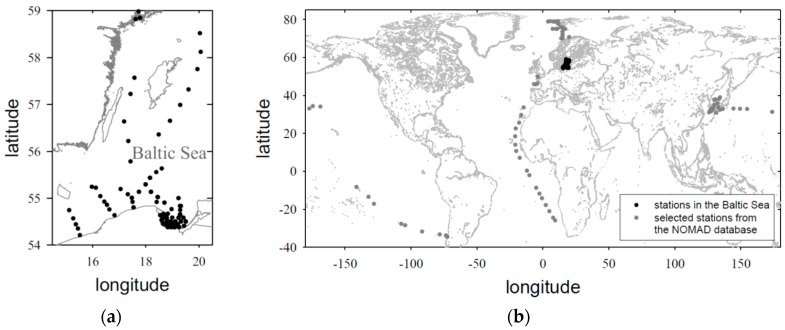
Sampling station locations: (**a**) stations in the southern and central Baltic Sea (authors’ own data); (**b**) stations in different regions of the global oceans (data from the NASA bio-Optical Marine Algorithm Dataset (NOMAD)).

The empirical formulas presented here were derived on the basis of combined data sets. We used our own original set of data obtained in the specific, optically complex environment of the Baltic Sea, and combined it with additional data that were available to us from the NASA bio-Optical Marine Algorithm Data set (NOMAD). The data obtained from the NOMAD database represent different regions of global oceans, mostly open waters. Figure 1 shows the different locations in which all the data used in this work were collected.

### 2.1. Baltic Sea Data Set

The authors’ original data set was gathered at 148 stations in the surface waters of the southern and central Baltic Sea, during 6 cruises of r/v “Oceania” in spring (April 2011, May 2013, 2014, 2015) and late summer (September 2011, 2012). The in situ optical measurements included spectral values of the light backscattering coefficient in seawater *b_b_*, the light absorption coefficient by all non-water constituents of seawater *a_n_* and the remote-sensing reflectance *R_rs_*. The methodology used for in situ measurements has already been described in our earlier papers (see e.g., [13,25]), but we recapitulate the most important details below.

#### 2.1.1. In Situ Optical Measurements

Optical measurements were carried out at each station according to a standardized protocol tailored to the capabilities of the vessel and available research equipment. For logistical reasons, two main instruments for measuring water IOPs—the HydroScat-4 spectral backscattering meter (HOBI Labs, Bellevue, WA, USA) and AC-9 spectral absorption-attenuation meter (WET Labs, Philomath, OR, USA)—were lowered simultaneously, but on separate frames, on the same side of the ship at a horizontal distance of about 20 m. After immersion in water, signal stability was checked and signals were measured for at least 60 sec as part of the so-called “surface measurements mode”, before standard profiling of the water column commenced. Both instruments were submerged to a minimum depth beneath the water surface to prevent the generation of air bubbles (in practice to a depth of about 1 m). This additional procedure of special measurements carried out in the surface layer was dictated, among other things, by our wish to be consistent with the simultaneous collection of discrete seawater samples for biogeochemical analysis (the relevant results have already been presented in [10,11,25]). For the purposes of this study, only signals recorded in “surface mode” were averaged (without filtering out spikes) and we did not take into account possible changes in sea water IOPs with depth.

The backscattering coefficient *b_b_*(*λ*) [m^−1^] was measured with the HydroScat-4 instrument at 4 wavelengths—420, 488, 550, 620 nm, using the methods described by Maffione and Dana [26,27]. To the averaged signals registered in subsurface water layer the standard method of correcting for the incomplete recovery of the light backscattered in highly attenuating waters was applied in accordance with User’s Manual [28] (the so-called sigma correction); additional data on absorption and attenuation measured with the AC-9 instrument were used for this purpose. To obtain values of light backscattering from suspended particles only, *b_bp_*(*λ*), theoretical values of the backscattering coefficient for pure water *b_bw_*(*λ*) were subtracted according to Morel [29].

The AC-9 instrument equipped with a 25 cm pathlength was used to measure the light absorption coefficient by all non-water (suspended and dissolved) constituents of seawater *a_n_*(*λ*) [m^−1^] at 9 wavelengths: 412, 440, 488, 510, 532, 555, 650, 676 and 715 nm. This instrument was integrated with a standard ctd probe and equipped with a pump and a flow-through system. The standard methods of corrections were applied to temperature- and salinity-dependent water absorption [30], and to the incomplete recovery of the scattered light in the absorption tube (the so-called proportional method) with the value of *a_n_*(715) assumed to be 0 [31]. Absorption coefficients for pure water *a_w_*(*λ*) were added (a combination of data from different sources [32,33,34]) in order to obtain values of *a*(*λ*) (total light absorption coefficient of seawater).

Radiometric measurements were carried out using the C-OPS compact optical profiling system (Biospherical instruments Inc.) at a distance of about 10–30 m from the ship to prevent shading. If possible, the radiometric measurements were carried out at the same time as the IOP measurements, or were started with a delay of no more than 15 min. The remote-sensing reflectance just above seawater *R_rs_*(*λ*) [sr^−1^] was calculated from radiometric measurements at 17 wavelengths from 340 to 765 nm. The following quantities were directly measured with the C-OPS instrument: the upward radiance profiles in water *L_u_*(*z*, *λ*) and the downward irradiance just above the water *E_d_*(0^+^, *λ*). Radiometers were equipped with tilt and roll sensors; only nadir measurements have been taken into account to minimalize uncertainty related to additional corrections of various angular effects in analysis of measurements of upward radiance. To estimate the upward radiance “just below the sea surface” *L_u_*(0^−^, *λ*), measurements of the profiles of upward radiance *L_u_*(*z*, *λ*) were extrapolated from a subsurface layer of 0.5–2 m using the attenuation coefficient for upward radiance *K_Lu_*(*z*, *λ*). The latter was calculated as the local slope of ln[*L_u_*(*z*, *λ*)] measured over a depth interval spanning a few metres in the surface layer. The thickness of this depth interval depended on the extent to which the surface layer was homogeneous (typically about 3 m). The correction for the self-shading effect in the upward radiance just below the sea surface *L_u_*(0^−^, λ) was also applied [35,36]. Then, the water-leaving radiance *L_w_*(0^+^, *λ*) was obtained from estimated *L_u_*(0^−^,*λ*), using a factor of 0.544 calculated from the “n^2^ law for radiance” (see e.g., [2]). Finally, the *R_rs_*(*λ*) was calculated as *L_w_*(0^+^, *λ*)/*E_d_*(0^+^, *λ*).

All our instruments for IOP and radiometric measurements were periodically calibrated. The HydroScat-4 was factory-calibrated every two years; calibration factors at different spectral channels differed on average by less than 4% between consecutive calibrations (<2% differences for the 620 nm channel). The AC-9 instrument was factory-calibrated every year; calibration factors between consecutive years differed by <3% (average) and ca 4% for 440 nm. The stability of the AC-9’s calibration was regularly checked by performing scans in ultrapure water and in air. The C-OPS system was likewise calibrated on a regular basis in a factory calibration facility (in most cases annually) and no significant deviations of the calibration coefficients were recorded at that time. Since we had two C-OPS systems at our disposal, we were able to regularly compare them and check the stability of the radiometric performance of our devices.

#### 2.1.2. Data Interpolation/Extrapolation

The in situ optical instruments we used at sea differed in both the number and location of available individual spectral bands. In order to conduct further quantitative analyses, we decided to interpolate (and in some cases to extrapolate) the data to the selected eleven wavelengths of light: 412, 440, 488, 510, 532, 555, 589, 620, 650, 676 and 715 nm. For this purpose, we performed linear interpolations of the values of coefficients *b_bp_*(*λ*) and *a_n_*(*λ*), as well as the reflectance *R_rs_*(*λ*). In these calculations, we used the closest pairs of available bands from the set of original measured data.

### 2.2. Additional Data From the NOMAD Database

The additional data used in this work to extend the ranges of variability of the quantities analysed were obtained from the publicly available NOMAD database. From this, we selected sets of optical data that included spectra of backscattering coefficients, and also downward irradiance and water-leaving radiance spectra with measurements performed, among others, in the red spectral channels (at either 619 nm or 625 nm). As a result, and after initial quality control, we were able to select 90 sets of data for which we performed similar simple linear interpolations to the same eleven spectral bands, as we had done in case of the Baltic Sea data (the selected sets of NOMAD data correspond to the cruises marked in this database as: *“rb-01-02”*, *“ant-xxiii-1”*, *“biosope 3”*, *“oceania 1998”* and *“oceania 1999”*). Twenty-five of these 90 cases included information on absorption coefficients.

### 2.3. Selected Aspects of the Quasi Analytical Algorithm (QAA)

When giving examples of new algorithms in this paper, we will refer to the original quasi-analytical algorithm (QAA), developed by Lee at al. [5]. All the details concerning this particular algorithm may be found, for example, in the documentation of its latest version (version 6 [37]); nevertheless, we recall here certain aspects that will be important for the later analysis.

The QAA, derived on the basis of data gathered mostly in the open waters of global oceans, combines a few simplified or purely empirical formulas with other fully analytical steps in the subsequent calculations. The input to this algorithm comprises the spectral values of the remote sensing reflectance just above the sea surface, *R_rs_*. Its first simplified or empirical steps involve:

- estimating the spectral values of the remote-sensing reflectance just below the sea surface, *r_rs_*, using the simplified relationship:
  *r_rs_*(*λ*) = *R_rs_*(*λ*)/[0.52 + 1.7 × *R_rs_*(*λ*)]; (1)
- estimating the ratio *u*(*λ*), from the reflectance *r_rs_*, based on the simplified best-fit relationship:
  *r_rs_*(*λ*) = *g*_0_*u*(*λ*) + *g*_1_[*u*(*λ*)]^2^, (2)
  where *u*(*λ*) represents the ratio of the backscattering coefficient *b_b_*(*λ*) to the sum of absorption *a*(*λ*) and backscattering *b_b_*(*λ*), i.e.,:
  *u*(*λ*) = *b_b_*(*λ*)/[*a*(*λ*) + *b_b_*(*λ*)], (3)
  and the best fit coefficients *g*_0_ and *g*_1_ are taken as equal 0.0895 and 01247, respectively (according to Lee et al. [5]);
- estimating the absorption coefficient for a selected spectral band *λ*_0_, either green (the closest available band to 555 nm) or red (670 nm), where the selection of *λ*_0_ depends on the magnitude of the reflectance *R_rs_*(670). The absorption coefficient *a*(*λ*_0_) can be estimated with one of the simplified empirical expressions which can generally be described as functions of reflectances *r_rs_*, i.e.,:
  *a*(*λ*_0_) = f(*r_rs_*(*λ*)). (4)
  These particular functions use a combination of blue, green and red *r_rs_* bands (for the sake of brevity, we do not give detailed formulas here; they can be found in the original QAA documentation [5,37]).

In the next step, after *a*(*λ*_0_) has been estimated, the QAA allows one to analytically calculate the backscattering coefficient *b_b_*(*λ*_0_), using the relationship given by Equation (3); and, by taking into account the known values of pure water backscattering, *b_bw_*(*λ*), the backscattering coefficient of particulate matter *b_bp_*(*λ*_0_) can also be calculated. The algorithm then assumes that the spectral shape of coefficient *b_bp_*(*λ*) can be described by the following power function:

*b_bp_*(*λ*) = *b_bp_*(*λ*_0_) [*λ*/*λ*_0_]^−*γ*^, (5)

which is a simplification often adopted in the practical analysis of optical data (see e.g., [8,38,39]). The slope parameter *γ* needed to apply the spectral shape assumed by Equation (5) is calculated using another empirical best-fit equation:

*γ* = 2[1 − 1.2exp(−0.9(*r_rs_*(443)/*r_rs_*(555))]. (6)

Then, with the help of Equation (5), the full spectrum of *b_bp_*(*λ*) can be calculated, and later, fully analytically (again using of Equation (3)), the full spectrum of *a*(*λ*) as well. The QAA includes more computational steps based on further empirical formulas. In these steps the total absorption coefficient *a*(*λ*) is divided into two components: one representing phytoplankton particles and the other representing the sum of absorption by detritus and by dissolved organic matter. In this paper, however, we will overlook these final steps.

At this point, it should be noted that the empirical steps of the QAA, represented above by Equations (4) and (6), are key elements of this algorithm. They are far-reaching simplifications. At these stages, based on selected features of the shape of the reflectance spectrum, “intelligent guesses” are made for the values of the absorption coefficient *a*(*λ*_0_) and the slope parameter *γ*. These steps are of major importance for the accuracy of all subsequent calculations, leading to the analytical retrieval of full spectra of absorption and backscattering coefficients. As we will show, in situations when concentrations of both suspended and dissolved matter in seawater are high, i.e., in conditions typical of the Baltic Sea, other empirical relationships may be considered as very preliminary computational steps in semi-analytical algorithms, similar to the classic QAA.

### 2.4. The Hue Angle

**Figure 2:**
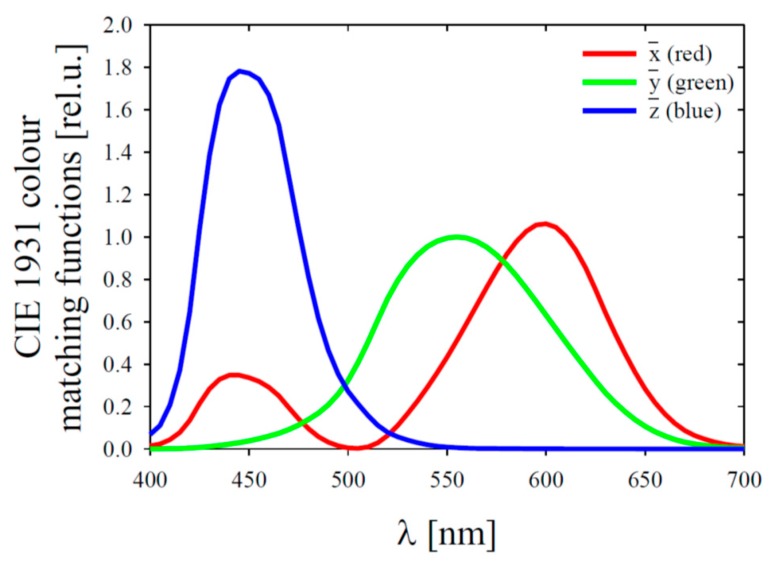
CIE 1931 colour matching functions.

Our analyses also took into account the hue angle (usually denoted by *α*). This is a single quantity which mathematically represents the sensation of water colour as it might be perceived by the human eye: it can be calculated directly from the spectral shape of *R_rs_* (see e.g., [16]). Generally, the human eye has three kinds of cone cells that sense light in different broad spectral bands. The sensitivity of the average human eye can be represented by so-called colour matching functions (CMFs) [40]. Figure 2 depicts standard colorimetric 2-degree CMFs, denoted by $\bar{x}$, $\bar{y}$ and $\bar{z}$. These functions can be used to calculate quantities called tristimulus values: X, Y and Z. This is done by integrating the product of each CMF and *R_rs_*(*λ*) over the whole visible light spectrum:

(7) $$X=\int_{400\;nm}^{700\;nm}R_{rs}\left(\lambda \right)\bar{x}\left(\lambda \right)d\lambda ;\;Y=\int_{400\;nm}^{700\;nm}R_{rs}\left(\lambda \right)\bar{y}\left(\lambda \right)d\lambda ;\;Z=\int_{400\;nm}^{700\;nm}R_{rs}\left(\lambda \right)\bar{z}\left(\lambda \right)d\lambda .$$

Having specified the tristimulus values, we can calculate quantities known as chromaticity coordinates, x, y and z:

(8) x=X/(X+Y+Z); y=Y/(X+Y+Z); z=Z/(X+Y+Z).

The first two of these coordinates, the independent ones *x* and *y*, allow one to plot what is called a chromaticity diagram. Such a diagram can numerically represent the human eye’s sensation of colour, regardless of the light intensity. A certain point in the chromaticity diagram, called the “white point” (with coordinates *x_w_* = *y_w_* = 1/3), represents the light that the human eye would treat as “colourless” (either as white or grey depending on the intensity). For any given point (*x*, *y*) in the chromaticity diagram, the sought-after value of the hue angle *α* is defined as the value in degrees of the angle between the segment connecting the “white point” with the given point (*x*, *y*) and the X axis. Thus, the hue angle can be calculated as:

*α*[in degrees] = (180/π)(atan2(*y* − *y_w_*,*x* − *x_w_*) mod 2π), (9)

where atan2 stands for a 2-argument arctangent function, which may be defined as follows:

(10) $$\mathrm{atan}2\left(y,x\right)=\{\begin{array}{c} 2\arctan\left(\frac{y}{\sqrt{x^{2}+y^{2}}+x}\right)\;if\;x>0\;or\;y\neq 0\\ \pi \;if\;x<0\;and\;y=0\\ undefined\;if\;x=0\;and\;y=0 \end{array}$$

### 2.5. Simple Models of Water Colour

When analysing the new empirical formulas derived in this work, we will also refer to simple water colour models. We will take into account two simple models. One, denoted as “model A”, is designed to represent various conditions that can occur in the Baltic Sea, generally belonging to the Case 2 water category according to the classification introduced by Morel and Prieur [8]. The second model, “model B”, is constructed in a similar way, but has to represent the conditions in Case 1 waters, where all the optically active constituents can be correlated with the concentration of chlorophyll *a*. Both these simple models calculate the quantity *u* (defined earlier by Equation (3)). Importantly, this quantity is then treated as a simple proxy for the spectral shape of the remote-sensing reflectance *R_rs_* (because it is well known that in the first approximation the reflectance *R_rs_*(*λ*) changes proportionally to the value of *u*(*λ*) [2,41]). Both models will also calculate the hue angle *α* on the basis of the spectra of *u*. This is done using Equations (7)–(10), with *u*(*λ*) inserted into the calculations instead of *R_rs_*(*λ*).

Model A will account for different concentrations of chromophoric dissolved organic matter (CDOM) and different concentrations of both organic and inorganic particulate matter. The total absorption coefficient *a* is assumed to be the sum of absorption coefficients for pure water, CDOM and particulate matter (*a* = *a_w_* + *a_g_* + *a_p_*), and the total backscattering coefficient *b_b_* is assumed to be the sum of backscattering for pure water and particulate matter (*b_b_* = *b_bw_* + *b_bp_*). The IOPs of pure water are taken from the literature (*a_w_*—a combination from [32,33,34]; *b_bw_* from [29]). The model results presented later will correspond to four different cases of CDOM absorption *a_g_*(*λ*). These spectra are modelled using the following simplified formula:

*a_g_*(*λ*) = *a_g_*(440) exp[−*S_g_*(*λ* − 440)]. (11)

**Figure 3:**
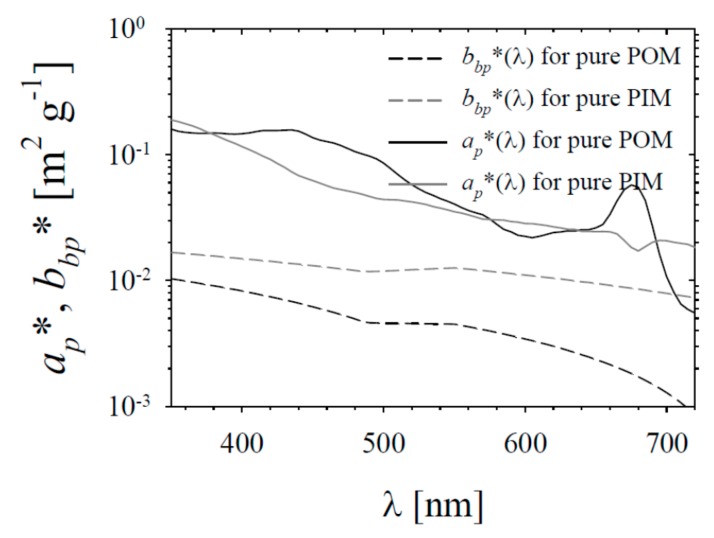
Assumed spectra of mass-specific absorption and backscattering coefficients, a_p_* and b_bp_*, for pure particulate organic matter (POM) and particulate inorganic matter (PIM) fractions, used by the simple water colour model adopted here to represent different conditions in the Baltic Sea (model A).

Four values of *a_g_*(440) will be taken into account: 0, 0.2, 0.7 and 1.6 m^−1^. The first of these values represent the hypothetical situation with no CDOM in the water, the second and third represent typically low and high values of *a_g_*(440) that might be encountered in the coastal areas of the southern Baltic Sea; the fourth is an instance of a very high CDOM concentration, which can occur when the waters of the River Vistula (Wisła) enter the Baltic Sea. In all cases, we assume the spectral slope *S_g_* to be 0.0196. The IOPs of particulate matter in our simple model will be parameterized with the concentrations of the organic and inorganic fractions of particulate matter (POM and PIM). Mass-specific coefficients, denoted by a_p_* and b_bp_*, were established for the pure POM and PIM fractions; their values are presented in Figure 3. Note that all the values assumed here are only examples taken for the purposes of performing simple and illustrative modelling. However, these values are based on the authors’ own new dataset acquired in different seasons of the year in a coastal location on the southern Baltic Sea. The b_bp_* and a_p_* spectra for the pure organic and inorganic fractions were calculated with a methodology similar to the one described by Woźniak et al. [25]. The calculations with model A will be performed for the following POM and PIM concentrations: 0, 0.1, 0.2, 0.5, 1, 2, 5, 10, 20, 50 and 100 g m^−3^.

The second model, model B, will calculate the ratio *u* and the hue angle α in a similar way to model A, but the important difference is how the water IOPs are defined. In model B, which is intended to represent Case 1 waters, both particulate backscattering coefficient *b_bp_* and the absorption coefficient of particulate and dissolved matter (*a_n_* = *a_p_* + *a_g_*) are parameterized with only one quantity - the concentration of chlorophyll *a* (Chl *a*). This is done according to the set of formulas known as the “new” IOPs model for Case 1, which is used, among others, in Mobley’s well-known Hydrolight code (see e.g., [42,43]). We will take the following Chl *a* values into account in our computations: 0, 0.03, 0.1, 0.3, 1, 3, 10, 30, and 100 mg m^−3^.

## 3. Results and Discussion

### 3.1. General Characterization of the Dataset

**Figure 4:**
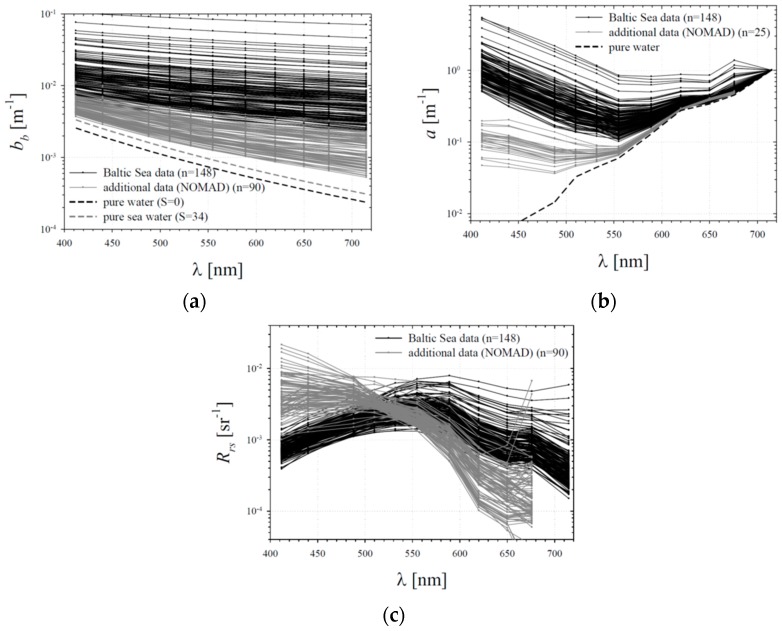
Empirical data analysed in this work: (**a**) spectra of the backscattering coefficient; (**b**) absorption coefficient; (**c**) remote-sensing reflectance. The authors’ original data from the Baltic Sea are shown as black curves; additional data from the NOMAD database are plotted in grey. Values representing pure water are plotted in panels a and b.

In general, the variability of different optical properties of water in our combined dataset is significant. The spectral backscattering and absorption coefficients of seawater measured in the Baltic Sea region alone are characterized by a variability of up to one order of magnitude (Figure 4a,b). Extending this set with data from NOMAD increases the overall range of variability to two orders of magnitude. Also, the variability of the remote-sensing reflectance is substantial. *R_rs_* spectra from the Baltic Sea generally show a maximum in the green region, and the changes in *R_rs_* values at longer wavelengths can be as high as one order of magnitude. The additional *R_rs_* data from NOMAD generally have different spectral shapes. Moreover, these *R_rs_* values are much higher in the blue range and much lower in the red range when compared to the Baltic Sea spectra. Overall, the variability of *R_rs_* for the combined dataset covers almost two orders of magnitude in the blue and red-light regions. (Figure 4c).

### 3.2. Empirical Relationships between the Backscattering Coefficient and the Remote-Sensing Reflectance

**Figure 5:**
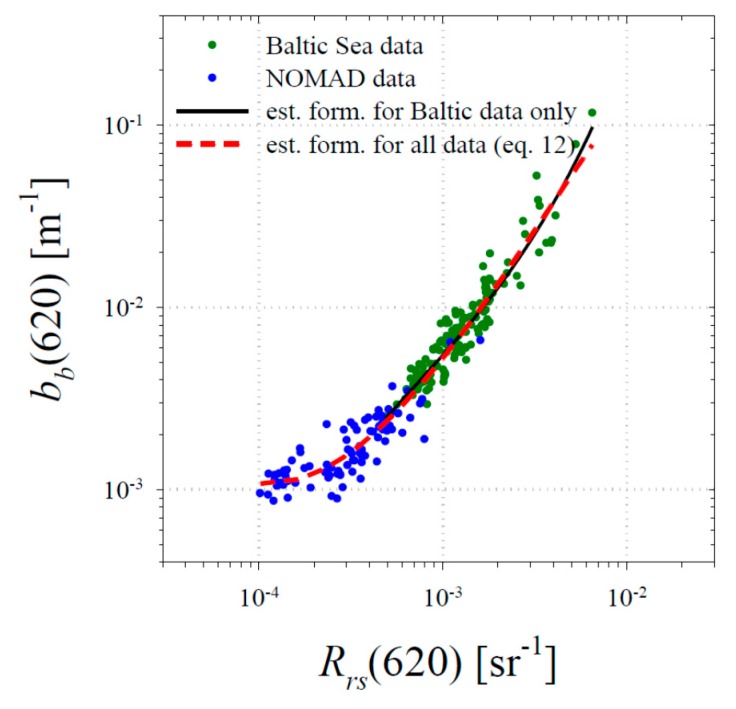
Relationship between the backscattering coefficient *b_b_* and the remote sensing reflectance *R_rs_* at 620 nm. Data from the Baltic Sea and the NOMAD database are shown by green and blue points. The two lines show the best-fit polynomial relationships.

Apart from the complex relationships that can theoretically occur between given apparent optical properties (radiances and irradiances on the basis of which the remote-sensing reflectance *R_rs_* is defined) and inherent optical properties describing light scattering and absorption by various components of seawater, here we performed statistical analyses of the dependences between measured values of *b_b_* and reflectance *R_rs_*. It turned out that, in contrast to the blue light range, approximate best-fit relationships between the logarithms of these two quantities could be derived for longer wavelengths of light, especially in the red. From the statistical point of view, the best relationships were found for the 620 nm band. These took the form of a third-order polynomial, which for the combined dataset (Baltic Sea and NOMAD data) is (see Figure 5):

log(*b_b_*(620)) = −0.206[log(*R_rs_*(620))]^3^ − 1.477[log(*R_rs_*(620))]^2^ − 2.029[log(*R_rs_*(620))] − 0.6384. (12)

The determination coefficient r^2^ for this relationship is 0.94. When we restricted the statistical analyses to the Baltic Sea data, a similar polynomial was found with a determination coefficient r^2^ of 0.90 (see the additional curve in Figure 5).

The fact that there is a statistical relationship between *b_b_* and *R_rs_*, described by Equation (12), can be explained by the existence of a chain of “component” relationships between quantities that are more directly related. In the red part of the spectrum, the backscattering coefficient *b_b_* strongly affects the overall value of the *u* ratio (*u* = *b_b_*/(*a* + *b_b_*)), since the total absorption *a* in this particular spectral region is dominated by absorption due to pure water. In turn, *u* is the quantity that strongly determines the value of the remote-sensing reflectance “just below” the sea surface, denoted as *r_rs_*. And finally, there must be a close relationship between *r_rs_* and the remote sensing reflectance “just above” the sea surface *R_rs_*. With our data, we were able to document two of these three relationships. For the combined data set, the best-fit statistical relationship between log(*b_b_*(620)) and log(*u*(620)) was found, which takes the following form:

log(*b_b_*(620)) = 0.4339[log(*u*(620))]^3^ + 2.502[log(*u*(620))]^2^ + 5.916[log(*u*(620))] + 2.803. (13)

**Figure 6:**
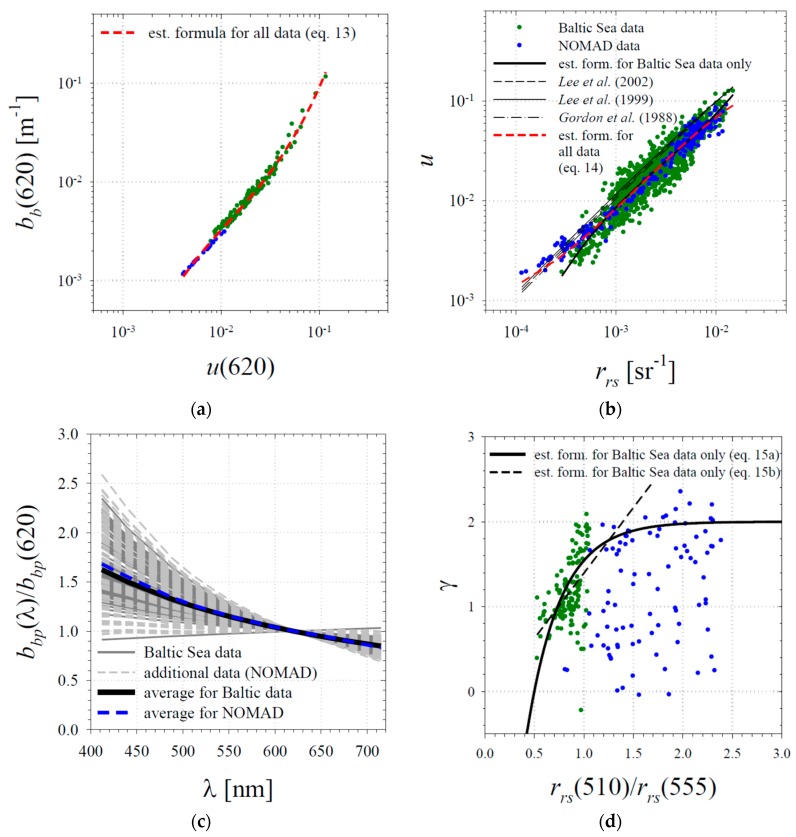
Selected relationships and estimated best-fit formulas: (**a**) the relationship between *b_b_*(620) and *u*(620); (**b**) the relationship between *u* and *r_rs_*; (**c**) spectra of the normalized backscattering coefficient *b_b_*(*λ*)/*b_b_*(620); (**d**) the relationship between the slope of the *b_bp_* coefficient spectrum *γ* and the reflectance ratio *r_rs_*(510)/*r_rs_*(555). In panel b, in addition to the line representing the best-fit relationships, selected formulas from the literature are also plotted (according to Lee et al. [5,44] and Gordon et al. [41]). Panel c shows spectra representing the average values of slope *γ* calculated for data from the Baltic Sea and from the NOMAD database.

This particular formula has a determination coefficient of r^2^ = 0.99. We were also able to find the best-fit relationship between log(*u*) and log(*r_rs_*). It takes the following form:

log(*u*) = −0.1116[log(*r_rs_*)]^3^ − 0.9328[log(*r_rs_*)]^2^ − 1.632[log(*r_rs_*)] − 1.59, (14)

with r^2^ = 0.90 (to obtain the latter relationship, all values of *u* and *r_rs_*, regardless of light wavelength, were taken into account). The formulas given by Equations (13) and (14) are plotted in Figure 6a,b. It should be noted here that Equation (14) differs to some extent from similar formulas often used in the subject literature (compare the additional curves plotted in Figure 6b representing the formulas given by Lee et al. [5,44] and Gordon et al. [41]). As regards the last statistical “component” relationship quoted, the one between the reflectances “just below” and “just above” the sea surface, *r_rs_* and *R_rs_*, we do not have the appropriate data available, and we are not in a position to present our own best-fit relationship. In further calculations, whenever necessary, we will use the simplified formula given by Lee et al. [5], cited earlier as Equation (1).

Using Equation (12), one can estimate the backscattering coefficient for wavelength 620 nm directly from the reflectance *R_rs_*. As we know, coefficient *b_b_*(*λ*) can be written as the sum of two coefficients: *b_bw_*(*λ*) representing the contribution of pure water and *b_bp_*(*λ*) representing the contribution of particulate matter. The values of *b_bw_*(*λ*) for pure water are known (in the subsequent calculations we take the values according to Morel [29]). If we assume that the spectral shape of coefficient *b_bp_*(*λ*) can be described by the power function given in Equation (5), and if we knew this function’s spectral slope *γ*, we could estimate the whole spectrum of *b_b_* coefficient from its value initially estimated in the red band. Figure 6c presents spectra of coefficient *b_bp_* approximated with power functions and normalized to the value for wavelength 620 nm. In the case of the Baltic Sea data, the average spectral slope *γ* is 1.16, while the values representing the 5th and 95th percentiles of *γ* are 0.65 and 1.9, respectively. In the case of the NOMAD data, the average *γ* is 1.21, and the 5th and 95th percentiles are 0.12 and 2.1, respectively. The original best-fit relationship used in QAA algorithm, given earlier as Equation (6), and using the 443 and 555 nm spectral bands to predict slope *γ*, in the case of the Baltic Sea data turned out to give strongly underestimated results. We also noticed that it relatively poorly described the weak trend existing in the subset of data obtained from the NOMAD database. Our analyses showed that better results could be achieved, at least in the case of the Baltic Sea data, if instead of the blue wavelength of 443 nm, we applied the longer wavelength of 510 nm. The best-fit formula retaining the mathematical form of Equation (6) that we found has the following form:

*γ* = 2[1 − 4.339exp[−2.943(*r_rs_*(510)/*r_rs_*(555))]] (15a)

The coefficient r^2^ for the linear regression between the expression log(1 − 0.5*γ*) and *r_rs_*(510)/*r_rs_*(555) in this case was 0.23. Alternatively, for Baltic data, it is possible to provide a best-fit formula using linear regression directly between *γ* and *r_rs_*(510)/*r_rs_*(555):

*γ* = 1.538(*r_rs_*(510)/*r_rs_*(555)) − 0.1456 (15b)

For this formula, r^2^ = 0.25. Low values of the determination coefficients r^2^ suggest, however, that both these formulas should be treated with caution, as they are very approximate (see Equation (15a,b) plotted in Figure 6d). We also tested other approaches, but they did not yield better results. We found, for example, that for our data there was no clear tendency between the slope *γ* and the magnitude of *b_bp_* at any wavelength, as opposed to the suggestion of Reynolds et al. [39].

### 3.3. Empirical Relationship between the Absorption Coefficient and the Hue Angle

**Figure 7:**
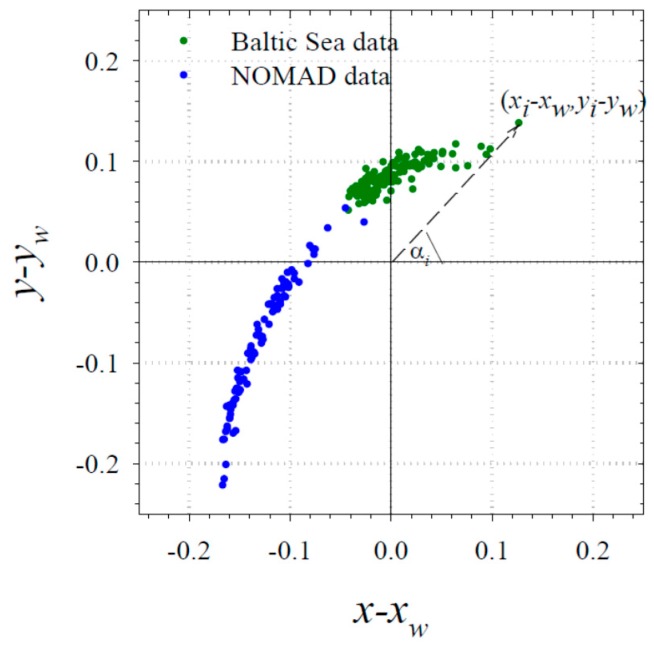
Plot of chromaticity coordinates calculated for data from the Baltic Sea and from the NOMAD database. The example of the hue angle *α* refers to one of the data points.

Another simple statistical relationship, which is of potential practical significance, is the one we found between the absorption coefficient and the sensation of colour perceived by the human eye. In Figure 7 we plotted the chromaticity coordinates calculated according to Equations (7) and (8) for all our available cases of *R_rs_* spectra, and already corrected by values representing the “white point”. These coordinates allowed the hue angle to be determined for each of the considered cases according to Equation (9).

**Figure 8:**
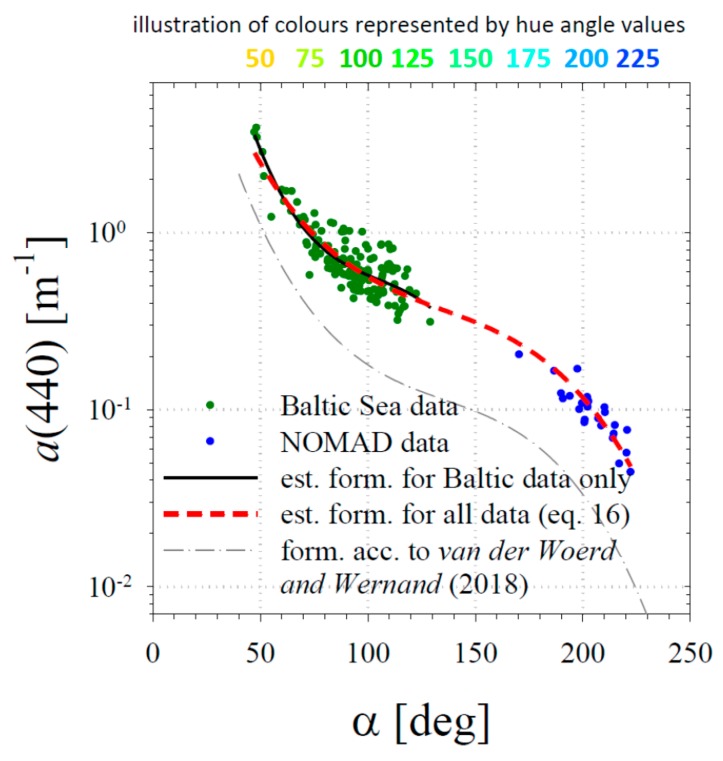
Relationship between the absorption coefficient *a*(440) and the hue angle α. Data from the Baltic Sea and the NOMAD database are shown as green and blue points. The two thick lines show the best polynomial relationships; the thin line represents the relationship according to van der Woerd and Wernand [22]. The additional legend above the horizontal axis illustrates the colours represented by selected values of the hue angle *α*.

We found that the calculated hue angles correlated well with the absorption coefficient, especially in the blue band of 440 nm (Figure 8). The best-fit formula found for the combined dataset takes the following form:

log(*a*(440)) = −7.406 × 10^−7^*α*^3^ + 2.999 × 10^−4^*α*^2^ − 0.04493*α* + 1.984, (16)

with the determination coefficient r^2^ = 0.93. A similar formula can also be found for Baltic Sea data alone (see the additional curve in Figure 8), but with a distinctly lower determination coefficient r^2^ of 0.76. A formula similar to ours, also in the form of a third-order polynomial, relating the logarithm of the absorption coefficient *a*(440) to the hue angle *α* has been suggested by van der Woerd and Wernand [22] (see Figure 8 in their work). We have drawn their formula in our Figure 8. It is generally similar in shape, but the *a*(440) values predicted for the same hue angles are generally 2–3 times smaller than ours. We will comment on this later, when we discuss the comparison of our formulas with simple modelling results.

### 3.4. Comparison of Empirical Formulas with the Results of Simple Modelling

**Figure 9:**
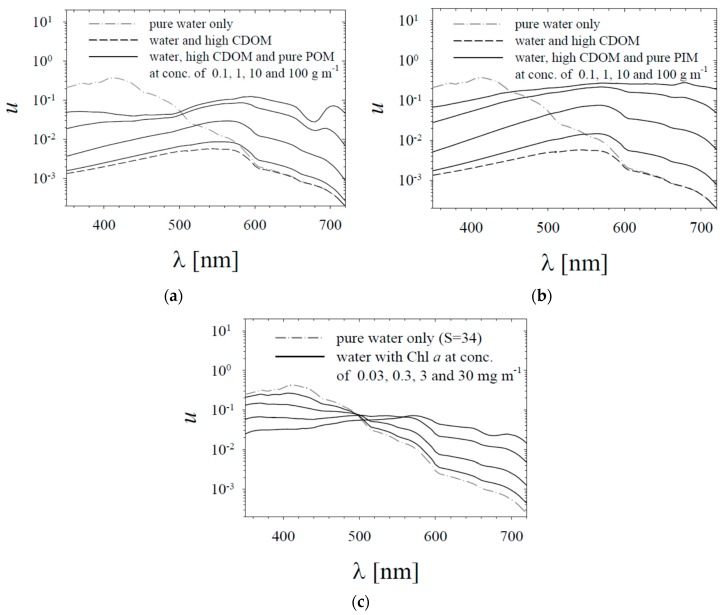
Example of results calculated using the simple models. Spectra of the *u* ratio (taken here as a simple proxy for the remote-sensing reflectance), calculated for: (**a**) model A and the case of high chromophoric dissolved organic matter (CDOM) concentration (assuming *a_g_*(440) = 0.7 m^−1^) and for different concentrations of the pure POM fraction; (**b**) as a, but for different concentrations of pure PIM fractions; (**c**) spectra calculated with model B for different chlorophyll *a* concentrations. On each panel, lines representing pure water are also plotted for reference.

The new formulas given by Equations (12)–(16) were found to be best-fit approximations of the relations observed between the empirical data. Below we compare some of these empirical formulas with results obtainable by simple theoretical modelling. As we mentioned in the previous section, we refer to two models: model A, designed to represent various conditions that may occur in Case 2 waters; and model B, representing conditions from Case 1 waters. For the sake of clarity, all of the results of model A shown below are plotted only for the cases where only pure POM or only pure PIM is present in the water. Examples of the *u* ratio spectra obtained with model A for high CDOM concentrations (with *a_g_*(440) = 0.7 m^−1^) and for various concentrations of pure POM and pure PIM are shown in Figure 9a,b. Examples of the *u* ratio spectra obtained with model B for selected concentrations of Chl *a* are illustrated in Figure 9c.

**Figure 10:**
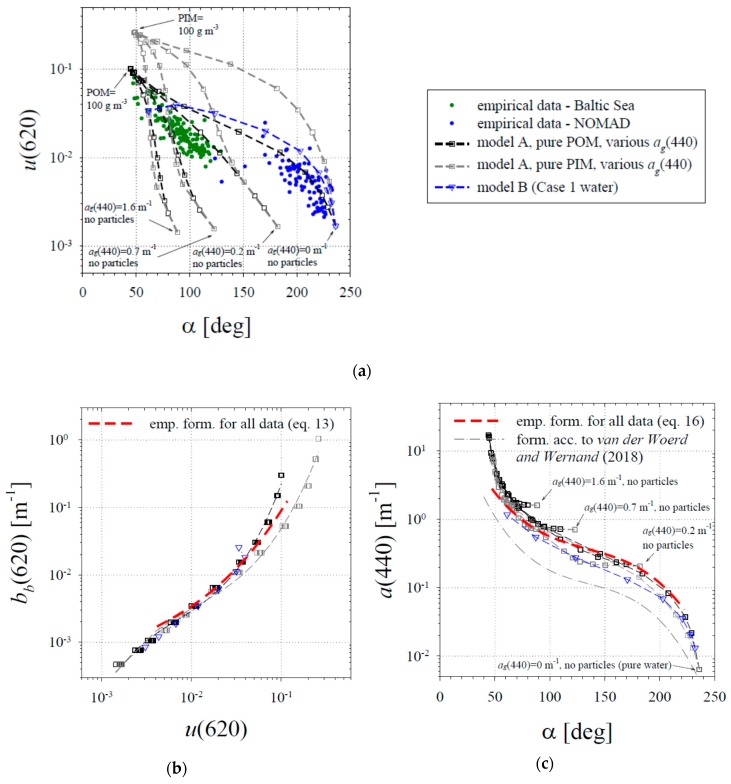
Comparison of empirical data and formulas with the results of simple modelling: (**a**) the relationship between the values of *u*(620) and the hue angle *α*; (**b**) the relationship between *b_b_*(620) and *u*(620); (**c**) the relationship between *a*(440) and the hue angle *α*. The green and blue points in panel a represent data from the Baltic Sea and from the NOMAD database, respectively. The empirical formulas (Equations (13) and (16)) are plotted in panels b and c. Model A results were used in all panels to draw various black and grey squares connected with the dashed curves. These curves represent different scenarios in which the CDOM concentration is assumed to be constant, whereas the concentration of the pure POM or PIM fraction ranges from 0 and 100 g m^−3^. The results of model B, representing different concentrations of chlorophyll *a* (from 0 to 100 mg m^−3^), were plotted in all the panels as blue triangles connected by a dashed blue line. Panel c also shows a curve representing the formula according to van der Woerd and Wernand [22].

The most important aspects of comparing the results obtained by modelling and the results of empirical data analysis are summarized in the three panels in Figure 10. Figure 10a compares the values of *u*(620) with the hue angles *α*. This diagram shows both modelled and empirical data. Different curves represent different scenarios of model A results, when the POM or PIM concentration changes, while *a_g_* is kept constant. As generally expected, the mutual positions of these curves may illustrate the trend that, with low and medium particulate matter concentrations and increasing absorption of CDOM, the hue angle can be clearly reduced, while the *u*(620) values remain more or less similar. Another generally expected modelling result is that at high particulate matter concentrations, *u*(620) may be clearly higher for pure PIM concentrations than for pure POM. Unlike the various scenarios represented by model A, the results of model B presented in this diagram form only one curve. Obviously, this is because this model uses a simplified IOP parameterization using only one variable—Chl *a*. If we look at the empirical data points shown in Figure 10a, we see that their positions seem to be broadly consistent with the ranges predicted by simple models. Points representing the Baltic Sea data lie mainly between the two modelled curves representing high and low *a_g_*(440) values for pure POM cases (note that for samples taken in the surface waters of the southern Baltic Sea, the average ratio of POM to (POM + PIM) is approximately 0.8, which means that particles in this basin are usually dominated by the organic fraction [10,25]). In the case of the empirical data points from the NOMAD database, some of them are close, but most of them are located slightly to the left of the curve representing the results of model B. This may be because the CDOM concentrations for the cases that we took from the NOMAD database were usually higher than those anticipated by the “new” IOPs Case 1 model.

The next panel, Figure 10b, illustrates the comparison of the empirical formula given by Equation (13) with the modelling results. First of all, it is worth noting that with the red wavelength considered, modelling results are, as expected, almost insensitive to different CDOM concentrations (the contribution of CDOM to the total absorption coefficient is marginal for this wavelength). The modelling results also show that regardless of the particulate matter composition in the range of low and medium backscattering values, we can expect a strong statistical relationship between *b_b_*(620) and *u*(620). Only for cases of high *b_b_*(620) do we observe that the model curves are separated between the extreme cases of pure POM and pure PIM. As we have already mentioned, suspended matter in Baltic surface waters is typically dominated by the organic fraction, so it seems reasonable that the line representing the empirical Equation (13) lies closer to the results of model A obtained for pure POM. Because the modelling results confirm the existence of a strong correlation between *b_b_*(620) and *u*(620), it also becomes an argument in favour of the empirical relationship between *b_b_*(620) and *R_rs_*(620) given by Equation (12). In turn, Figure 10c compares the modelling results with the empirical relationship given by Equation (16). Here, we can see that with three orders of magnitude of changes in *a*(440), there is a maximum of twofold differences in the modelled results for different scenarios giving the same hue angles. The largest deviations from the general trend formed by the modelled data can be observed only for hypothetical situations where no particulate matter was assumed to be present in the seawater, i.e., only high concentrations of CDOM. As we can see, the curve representing the empirical Equation (16) shows quite a similar trend to the one emerging from all the modelling results obtained from Case 2 waters. In this context, the aforementioned additional formula, taken from the work of van der Woerd and Wernand [22], also shows smaller predicted values of *a*(440) in comparison with the modelling results. To conclude, we consider that both comparisons with the results of modelling presented in Figure 10b,c provide additional arguments in favour of the empirical formulas presented in this article as practical solutions for estimating selected IOPs from *R_rs_* spectra.

### 3.5. Preliminary Assessment of Measurement Error Propagation

The main formulas presented in this work were derived on the basis of empirical data, which to some extent must have been encumbered with measurement errors. However, the statistical nature of these formulas (best-fit relationships obtained on large sets of data points), means that the impact of statistical errors on our original data should have been largely cancelled out. Only possible systematic errors (biases) could have affected the formulas. Assuming, however, that no significant biases occurred during derivation of these formulas, a separate problem is how their application in practice may propagate measurement errors contained in new data sets being analysed. Below we give examples of estimates that allow us to partially address this last problem.

**Figure 11:**
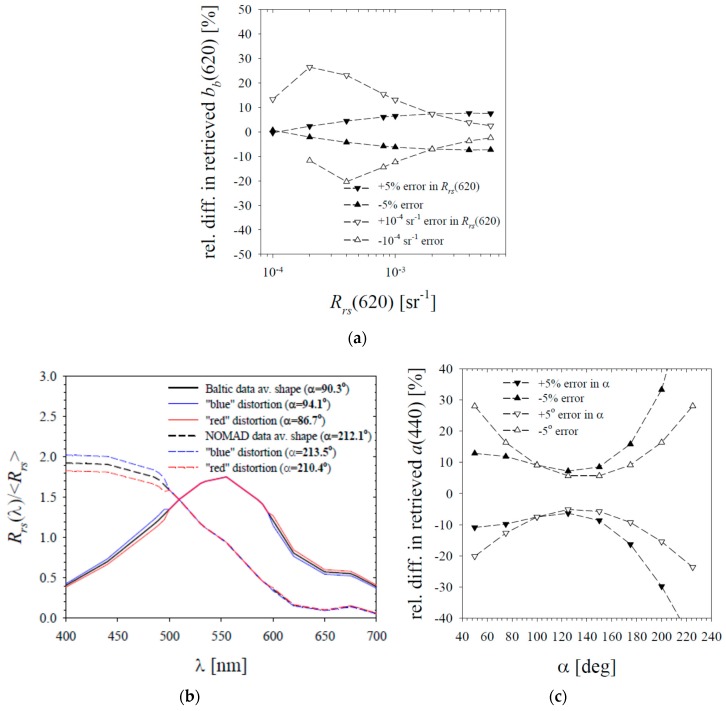
Results of calculation illustrating the discussion of the possible measurement error propagation related to the practical use of the empirical formulas presented here: (**a**) the relative differences in the retrieved values of *b_b_*(620) using Equation (12), calculated for the scenarios of different relative or absolute errors introduced in the input values of *R_rs_*(620); (**b**) average spectra of normalized reflectances *R_rs_*(*λ*)/<*R_rs_*> for the Baltic Sea data and NOMAD data, and their “distorted” versions; the calculated values of the hue angle *α* are given in the caption; (**c**) the relative differences in the retrieved values of *a*(440) with use of Equation (16), calculated for different relative or absolute errors introduced in the input values of the hue angle *α*. The relative differences in panels a and c were calculated according to the following formula: (rel. diff. in retrieved quantity *Y*)[%] = 100*[f(*X* + *ε*) − f(*X*)]/f(*X*), where *Y*—a dependent variable (either *b_b_*(620) or *a*(440)), X—an independent variable (either *R_rs_*(620) or *α*), f(X)—an empirical formula tested (either Equation (12) or Equation (16)), *ε*—an error introduced in the independent variable.

In the case of the formula enabling coefficient *b_b_*(620) to be calculated on the basis of *R_rs_*(620) (Equation (12)), one can make a relatively uncomplicated estimate. We considered two simplified scenarios. In the first, we assumed that the input data would be affected by an arbitrary statistical error, which is always +/−5% of the *R_rs_*(620) value. In the second such scenario, we assumed that the input data would always be burdened with an error of +/−10^−4^ sr^−1^. The first of these values may represent the generally desired accuracy sought in remote sensing studies, while the second one is a top-down estimate of what accuracy can be achieved in practice in red light bands in relation to global satellite research (see e.g., [45]). In the first scenario, the relative differences (representing relative errors) of the estimated coefficient *b_b_*(620) never exceed +/−8%, while in the second scenario, relative differences no greater than +/−23% occur for *R_rs_*(620) > 6 × 10^−4^ sr^−1^, and no greater than +/−15% occur for *R_rs_*(620) > 8 × 10^−4^ sr^−1^ (see Figure 11a). This means, as expected, that the proposed formula seems to be predestined for use in waters rich in suspended substances (such as Baltic waters), where the reflectance in the red band reaches values of 10^−3^ sr^−1^ and higher.

In the case of the second main formula that we propose, a comprehensive discussion of possible error propagation would be much more complicated and would probably exceed the scope of this paper. Nonetheless, we provide an example estimate which, in our opinion, allows at least a qualitative assessment of the problem.

The empirical formula given by Equation (16) uses the hue angle *α*, a quantity calculated from the full spectrum of *R_rs_*(*λ*). Errors occurring in different bands of the measured reflectance, which may generally be uncorrelated with each other, can affect the calculated value of the hue angle. To illustrate this, we take two typical spectral shapes, corresponding to the Baltic data set and the data from the NOMAD database. By typical spectral shapes we mean here the average values of normalized spectra denoted by *R_rs_*(*λ*)/<*R_rs_*> (in each case normalization was carried out to the average value of the reflectance from the entire spectrum, denoted by <*R_rs_*>; the input spectra used in calculations were the spectra analysed in this paper linearly interpolated every 5 nm). These averaged shapes *R_rs_*(*λ*)/<*R_rs_*> are shown in Figure 11b. In addition, two types of modification were introduced into each of these shapes to illustrate possible spectral “distortions” that could change their “effective colour”. The first distortion was to increase the value in the blue light range by 5% (from 400 to 500 nm), and at the same time to reduce the value in the red range by 5% (from 600 to 700 nm). In contrast, the second modification consisted of a corresponding decrease in the blue range and increase in the red range. Then, for each of these spectra, the hue angle *α* values were calculated (according to formulas 7 to 10, and taking the normalized shape *R_rs_*(*λ*)/<*R_rs_*> as the equivalent of the reflectance spectrum *R_rs_*(*λ*)). These values are given in the caption to Figure 11b. In the case of a typical Baltic shape we found *α* = 90.3˚, and after taking into account the distortions, we found relative differences of about +/−4%, what in terms of absolute values was about +4˚ and −3˚. For a typical shape of the data from the NOMAD database we found *α* = 212.1˚, and after taking into account the distortions the differences in percentages were smaller (below +/−1%), and in terms of absolute values ranged from about +1˚ to −2˚. These examples show that one can imagine hypothetical distortions of reflectance spectra caused by errors in various parts of the spectrum that could lead to noticeable changes in the value of the calculated hue angle. In the next step, we assume arbitrarily possible hue angle errors up to +/−5% or +/−5˚.

Figure 11c illustrates the impact that errors in the hue angle assessment of +/−5% or +/−5˚ may have on the retrieved value of the absorption coefficient *a*(440) using Equation (15). Both scenarios indicate that errors in the assessment of *a*(440) will not exceed +/−17% in the *α* range from about 75˚ to 175˚. The biggest errors may occur in very clean waters (for u > 200˚). The above statements, however, should probably be treated with some caution, and as qualitative rather than quantitative.

### 3.6. Potential Applications: an Example of a New Semi-Analytical Algorithm for IOP Retrieval

We can suggest at least a few potential applications of the new empirical formulas derived in this work. For fully professional applications, such as satellite optical data analysis, we can generally suggest that the estimated information about *b_bp_*(620) and *a*(440) can be used at least to perform the initial selection of *R_rs_* spectra into subgroups that clearly differ in their optical properties. In our opinion, this would help in the further development of various classification-based methods. The results shown in Figure 10a suggest that another possibility would be to use the hue angle *α* directly in combination with the magnitude of *R_rs_* in the red part of the spectrum, in order to distinguish different cases of seawater composition. As for applications that go beyond the fully professional ones, it seems that the formula binding the absorption coefficient with the water colour observed by the human eye (Equation (16)) may be relevant for the recently spreading Secchi disc and Forel-Ule scale applications within citizen science (see e.g., [13,16,17]). In addition to these general suggestions, we present two specific examples, showing how these new formulas can be used as calculation steps in new variants of semi-empirical spectral deconvolution algorithms for analysing *R_rs_* spectra. The first of these examples is discussed below, while the second, alternative example, is presented in Appendix A.

Table 1 shows a new algorithm, which is constructed in a similar way as the aforementioned standard QAA. It enables one to estimate the spectra of backscattering and absorption coefficients in the entire visible spectral range. In this example we propose to start the calculation by estimating the backscattering coefficient *b_b_*(620) (step 1), the full spectrum of the *u* ratio (step 2) and the absorption coefficient *a*(440) (step 3). This can be done by means of the empirical equations established in this work, i.e., 12, 14 and 16, as well as 7 to 10 for calculating the hue angle *α*. Later, from these empirically estimated quantities, the full spectra of coefficients *b_b_* and *a* can be retrieved in an analytical manner (steps from 4 to 7).

**Figure 12:**
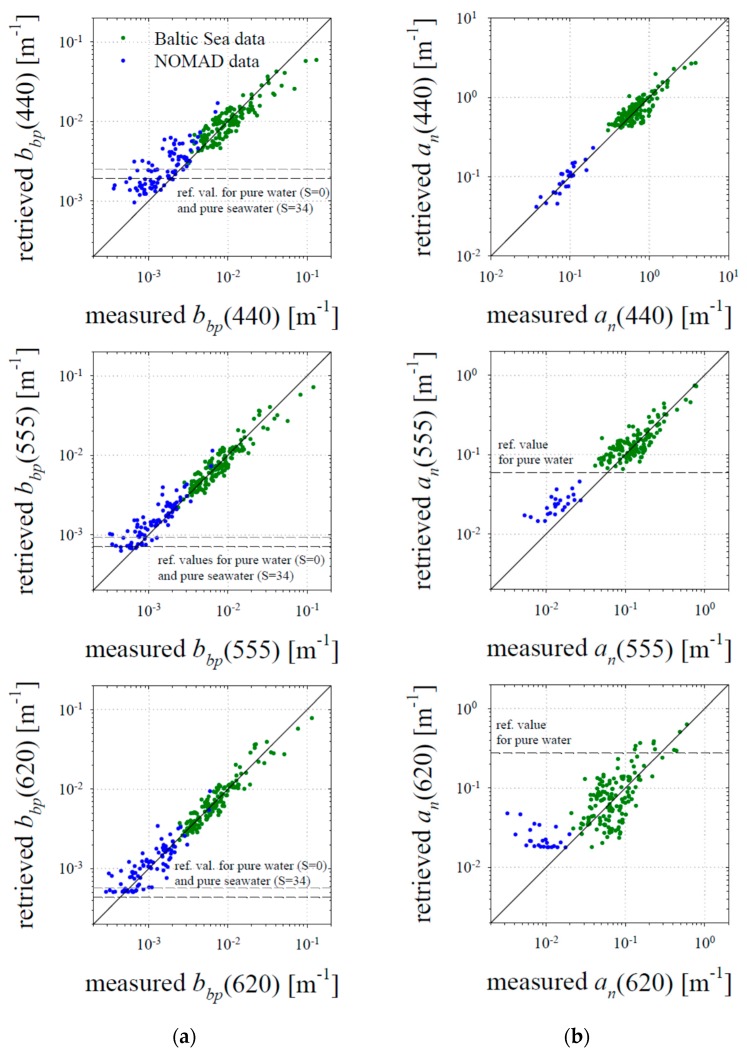
Comparison of optical coefficients retrieved using the new algorithm with the measured values for three spectral bands: 440, 555 and 620 nm: (**a**) comparison for the backscattering coefficient of particulate matter *b_bp_*; (**b**) comparison for the absorption coefficient of the sum of dissolved and suspended constituents *a_n_*.

The optical coefficients retrieved with the aid of this new algorithm at three wavelengths—440, 555 and 620 nm—are compared with the measured values in Figure 12, and Table 2 gives the details of the estimation errors calculated according to both standard arithmetic statistics and logarithmic statistics. Since the variability of the optical coefficients analysed here is more than two orders of magnitude, we will focus on the latter values only. In general, in the case of our entire dataset (i.e., combined data from the Baltic Sea and the NOMAD database), the new algorithm allows us to retrieve coefficients *b_bp_* with relatively low systematic errors: from −4.7% to 21.4% (this range represents 11 spectral bands analysed between 412 nm and 715 nm). The statistical error according to logarithmic statistics can be described by a quantity known as the standard error factor. This quantity allows us to estimate the statistical error range by dividing and multiplying by its value (see the footnote to Table 2). In the case of the new algorithm and coefficient *b_bp_*, the standard error factors range from 1.32 to 1.64. As generally expected, the precision in retrieving *b_bp_* with our algorithm is greater for data from the Baltic Sea, representing generally higher values of this quantity. When estimating coefficient *a_n_*(*λ*), we observe a general tendency to achieve greater precision for shorter wavelengths and poorer precision for longer wavelengths. This behaviour is also generally expected. The new algorithm estimates the total absorption coefficient a with an accuracy similar at all wavelengths (with standard error factors no higher than 1.30). However, when calculating coefficient *a_n_*, the contribution of pure water to coefficient *a* should be subtracted. Naturally, the estimation errors of coefficients *a_n_* are much larger in the spectral range where the absorption of pure water dominates the total absorption. In the range between 412 and 620 nm, the new algorithm retrieves *a_n_*(*λ*) with systematic errors from −18.8% to 14.2% and with standard error factors between 1.26 and 1.96. The standard error factors are relatively low, of the order of 1.43 or less, only in the spectral range between 412 to 555 nm, where the influence of pure water absorption is limited. Importantly, however, the accuracy of the new algorithm, though far from perfect, is clearly better than the results that can be obtained when the standard QAA is applied to our data (see the additional rows in Table 2 related to the use of the latest version of QAA). The application of QAA to our dataset leads to systematic errors ranging from approximately +30% to +50% for *b_bp_*, and from ca. −10% to −20% for *a_n_*. The standard error factors are also clearly higher compared to the results obtained using the new algorithm.

Clearly, the results discussed above and presented in Table 2 should not be treated as a formal validation of the new algorithm. For such a purpose an additional, independent set of data not used in formulating the new algorithm should be used. But all these results point to the fact that for data representing a marine environment with high concentrations of both particulate and suspended matter, one can use relationships other than the standard empirical ones in the formulation of the semi-analytical inversion algorithm. In our opinion, the new algorithm, alternative to the standard QAA, could be used at least in situations where *R_rs_*(620) is equal to or greater than ca. 7 × 10^−4^ sr^−1^, which corresponds to the lower range (5th percentile) of the values that we recorded in the Baltic Sea.

## 4. Summary

The empirical formulas presented in this work were derived on the basis of a combined dataset. Among others things, we used our own set of data obtained in the specific, optically complex environment of the Baltic Sea, which is characterized by high concentrations of both dissolved and suspended matter. To increase the potential applicability of these formulas, this dataset was expanded with additional data from the NOMAD database, representing different regions of the global oceans, with generally much lower concentrations of optically important water constituents.

One of the new empirical formulas presented (Equation (12)) can be used to estimate the backscattering coefficient *b_b_*(620) directly from the magnitude of the remote-sensing reflectance *R_rs_* in the red part of the visible spectrum. Its existence can be explained by the chain of more direct relationships between the backscattering coefficient *b_b_* in the red band, the ratio of backscattering to the sum of absorption and backscattering (*u*), and the remote-sensing reflectances “just below” and “just above” the sea surface (*r_rs_* and *R_rs_*, respectively).

Another empirical formula presented in this work, is the one which relates the absorption coefficient *a*(440) to the hue angle *α* (Equation (16)). The latter is a single quantitative measure of the colour of water as it might be perceived by the human eye, or by any hypothetical radiometric instruments having three independent broad channels for acquiring colour information (i.e., blue, green and red channels). The hue angle *α* can be easily calculated if the spectral shape of *R_rs_* is known.

Comparison of the new empirical formulas with the results of simple modelling performed for both Case 1 and Case 2 scenarios appears to confirm that they can be treated as acceptable first approximations of the dependences between remote-sensing reflectance and water IOPs in selected spectral bands.

One possible application of the new formulas is to use them as first calculation steps of the new version of the semi-analytical algorithm for analysing remote-sensing reflectance spectra (see algorithm in Table 2). This algorithm can offer an alternative to the known, standard quasi-analytical algorithm (QAA). It enables one to estimate the full spectra of the backscattering and absorption coefficients in the entire visible spectral range, and it does not require any additional a priori assumptions regarding the spectral shape of the absorption coefficients of dissolved and suspended seawater components. When applied to our dataset, the new algorithm offers a better precision than the current version of QAA. We believe that semi-empirical algorithms of this type, belonging to the category described as spectral deconvolution, should be developed further. They should increase the precision of IOP retrieval in different marine environments, where there may be significant changes in the optical properties of various components of seawater, and where, for example, the assumption of the constant/invariant shape of the spectral absorption coefficient of phytoplankton should be avoided.

It also seems to us that, in addition to being used as an element in multi-stage algorithms, the new formula linking the absorption coefficient with the hue angle may be of particular interest to the marine optics research community. In recent years, the trichromatic color vision mechanism has been used in practice only sporadically, and in our opinion, it has great potential both in purely professional applications and for the development of citizen science.

## Figures and Tables

**Table 1:** An example of the new semi-analytical algorithm.

NEW ALGORITHM
1. ***b_b_*(620) = f(*R_rs_*(620))** (emp. formula—Equation (12))
2. ***u*(*λ*) = f(*r_rs_*(*λ*))** (emp. formula—Equation (14)) where *r_rs_*(λ) calculated acc. to Lee et al. (2002) (Equation (1))
3. ***a*(440) = f(*α*)** (emp. formula—Equation (16) where *α* = f(*R_rs_*(*λ*)) (Equations (7)–(10))
4. *b_b_*(440) = [*a*(440)*u*(440)]/[1 − *u*(440)]; *b_bp_*(440) = *b_b_*(440) − *b_bw_*(440)
5. *γ* = log[*b_bp_*(440)/(*b_b_*(620) − *b_bw_*(620))]/log [620/440])
6. *b_bp_*(*λ*) = [*b_b_*(620) − *b_bw_*(620)] [*λ*/620]^-*γ*^; *b_b_*(*λ*) = *b_bw_*(*λ*) + *b_bp_*(*λ*))
7. *a*(*λ*) = *b_b_*(*λ*)/[(1/*u*(*λ*)) − 1]; *a_n_*(*λ*) = *a*(*λ*) − *a_w_*(*λ*)

**Table 2:** Summary of estimation errors of coefficients *b_bp_* and *a_n_* obtained when the new algorithm (formulated in this work) and the standard quasi-analytical algorithm (QAA) (according to Lee et al. [5]; version 6 [37]) were applied to the entire dataset used in this work, as well as to the subsets representing the Baltic Sea data. The statistical parameters calculated according to arithmetic statistics: the mean normalized bias (*MNB*) ^1^ and the normalized root mean square error (*NRMSE*) ^2^; the parameters calculated according to logarithmic statistics: the systematic error (*sys.err.*) ^3^ and the standard error factor (*X*) ^4^.

Retrieved Quantity		NEW ALGORITHM			QAA v6		
	Wavelength	440 nm	555 nm	620 nm	440 nm	555 nm	620 nm
***b_bp_***	*MNB* [%]	30.1	11.0	5.1	72.4	75.1	77.6
**(all data)**	*NRMSE* [%]	67.2	36.4	32.2	393.9	358.9	341.9
**(*n* = 238)**	***sys. err.* [%]**	**17.5**	**6.2**	**0.9**	**29.2**	**41.4**	**47.2**
	***X***	**1.54**	**1.34**	**1.32**	**1.72**	**1.55**	**1.49**
***b_bp_***	*MNB* [%]	−0.3	−0.4	0.1	3.4	23.6	34.9
**(Baltic Sea)**	*NRMSE* [%]	29.8	24.2	23.5	29.9	31.1	32.1
**(*n* = 148)**	***sys. err.* [%]**	**−4.7**	**−3.2**	**−2.5**	**−0.8**	**19.8**	**31.2**
	***X***	**1.36**	**1.28**	**1.26**	**1.33**	**1.28**	**1.27**
***a_n_***	*MNB* [%]	2.8	21.7	47.3	−19.2	−5.7	29.5
**(all data)**	*NRMSE* [%]	22.6	46	149.5	20.2	35.2	104.1
**(*n* = 173)**	***sys. err.* [%]**	**0.2**	**14**	**14.2**	**−21.8**	**−12.4**	*n.a.*
	***X***	**1.26**	**1.43**	**1.96**	**1.3**	**1.48**	*n.a.*
***a_n_***	*MNB* [%]	2.5	12.9	16	−18.4	−2.7	32.3
**(Baltic Sea)**	*NRMSE* [%]	23.1	38.9	66	21	35.1	97.7
**(*n* = 148)**	***sys. err.* [%]**	**−0.2**	**6.9**	**−0.7**	**−21.2**	**−9.1**	***n.a.***
	***X***	**1.26**	**1.39**	**1.76**	**1.31**	**1.46**	***n.a.***

^1^$MNB=\frac{1}{n}\sum_{i=1}^{n}\left(\frac{P_{i}\;-\;O_{i}}{O_{i}}\right)$, where *P_i_*, *O_i_*—predicted and observed values, respectively;

^2^$NRMSE={\left[\frac{1}{n\;-\;1}\sum_{i=1}^{n}{\left(\frac{P_{i}\;-\;O_{i}}{O_{i}}\;-\;MNB\right)}^{2}\right]}^{\frac{1}{2}}$,

^3^$sys.err.=10^{\langle \log\left(\frac{P_{i}}{O_{i}}\right)\rangle }\;-\;1;\;\langle \log\left(\frac{P_{i}}{O_{i}}\right)\rangle =\frac{1}{n}\sum_{i=1}^{n\;}\log\left(\frac{P_{i}}{O_{i}}\right)$;

^4^ $X=10^{\sigma_{log}};\;\sigma_{log}={\left[\frac{1}{n\;-\;1}\sum_{i=1}^{n}{\left(\log\left(\frac{P_{i}}{O_{i}}\right)\;-\;\frac{1}{n}\sum_{j=1}^{n}\log\left(\frac{P_{i}}{O_{i}}\right)\right)}^{2}\right]}^{\frac{1}{2}};$

*X* allows one to quantify the range of the statistical error, which extends from the value of *σ*_−_ = (1/*X*) − 1 to the value of *σ*_+ _= *X* − 1.

## Appendix A

Table A1 shows an alternative version of the new algorithm that can be constructed in a similar way as the standard QAA. In this alternative version, we propose to start the calculation by estimating the backscattering coefficient *b_b_*(620) (step 1), the full spectrum of the *u* ratio (step 2) and the spectral slope of the suspended matter backscattering coefficient *γ* (step 3). This can be done by means of the empirical equations derived in this work, i.e., 12, 14 and 15a. Later, from these empirically estimated quantities, the full spectra of coefficients *b_b_* and *a* can be retrieved in an analytical manner (steps 4 and 5). Please note that this version does not use an empirical formula linking the absorption coefficient *a*(440) with the hue angle *α*. The estimation errors for three wavelengths—440, 555 and 620 nm—are given in Table A2.

**Table A1:** An alternative example of the new semi-analytical algorithm.

ALTERNATIVE NEW ALGORITHM
1. ***b_b_*(620) = f(*R_rs_*(620))** (emp. formula—Equation (12))
2. ***u*(*λ*) = f(*r_rs_*(*λ*))** (emp. formula—Equation (14)) where *r_rs_*(*λ*) calculated acc. to Lee et al. (2002) (Equation (1))
3. ***γ* = f(*r_rs_*(510)/*r_rs_*(555))** (emp. formula—Equation (15a))
4. *b_bp_*(*λ*) = [*b_b_*(620) − *b_bw_*(620)] [*λ*/620]^−*γ*^; *b_b_*(*λ*) = *b_bw_*(*λ*) + *b_bp_*(*λ*)
5. *a*(*λ*) = *b_b_*(*λ*)/[(1/*u*(*λ*)) − 1]; *a_n_*(*λ*) = *a*(*λ*) − *a_w_*(*λ*)

**Table A2:** Summary of estimation errors of coefficients *b_bp_* and *a_n_* obtained when the alternative version of the new algorithm were applied to the entire dataset used in this work, as well as to the subsets representing the Baltic Sea data. The statistical parameters are the same as in Table 2.

Retrieved Quantity		ALT. NEW ALG.		
	Wavelength	440 nm	555 nm	620 nm
***b_bp_***	*MNB* [%]	19.8	9.4	5.1
**(all data)**	*NRMSE* [%]	52.1	36.5	32.2
**(*n* = 238)**	***sys. err.* [%]**	**11.9**	**4.5**	**0.9**
	***X***	**1.42**	**1.34**	**1.32**
***b_bp_***	*MNB* [%]	4.2	1.2	0.1
**(Baltic Sea)**	*NRMSE* [%]	29.7	24.6	23.5
**(*n* = 148)**	***sys. err.* [%]**	**0.1**	**−1.7**	**−2.5**
	***X***	**1.33**	**1.28**	**1.26**
***a_n_***	*MNB* [%]	6.6	24	47.3
**(all data)**	*NRMSE* [%]	23.3	47.7	149.5
**(*n* = 173)**	***sys. err.* [%]**	**4**	**16.2**	**14.2**
	***X***	**1.25**	**1.43**	**1.96**
***a_n_***	*MNB* [%]	7	15	16
**(Baltic Sea)**	*NRMSE* [%]	23.6	40.5	66
**(*n* = 148)**	***sys. err.* [%]**	**4.2**	**8.9**	**−0.7**
	***X***	**1.26**	**1.39**	**1.76**
